# Mamoona Rana, MA, MBBS

**DOI:** 10.1192/bjb.2021.10

**Published:** 2021-06

**Authors:** Afifa Qazi

Psychiatric trainee, North East London NHS Foundation Trust, UK

**Figure:**
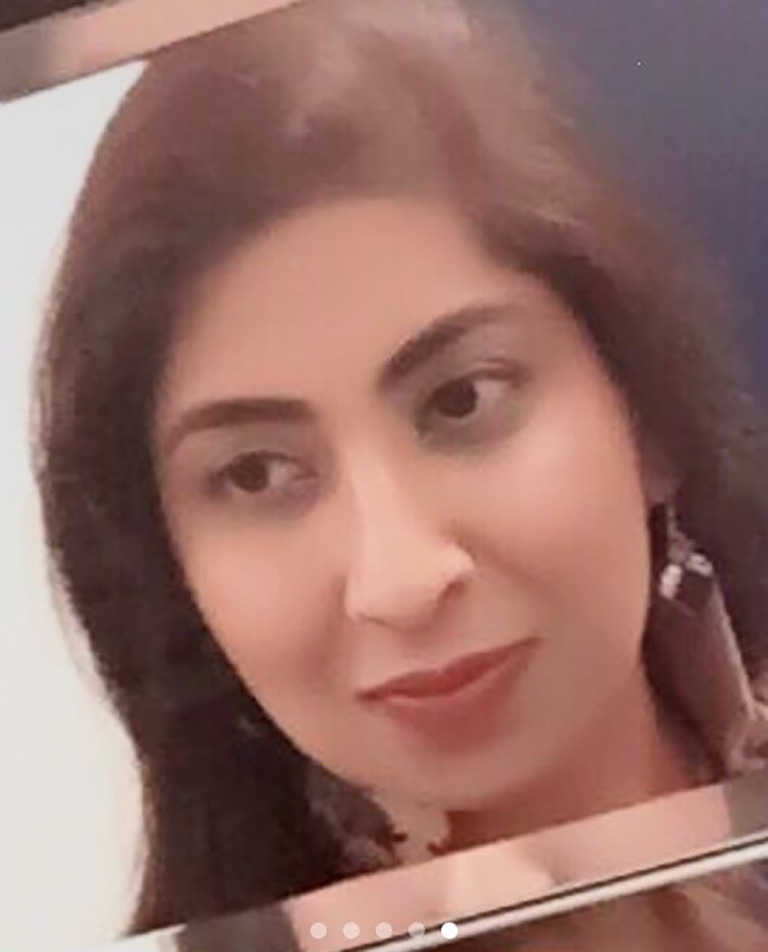

Dr Mamoona Rana, a much loved, highly regarded, enormously valued and committed junior psychiatrist, died at the age of 48 years of COVID-19 on 16 April 2020 after a short illness.

Mamoona was born in Punjab, Pakistan, the youngest of four children. She graduated from Lahore Medical College, worked in her native country for some years, after which she and her husband emigrated to the UK. She secured a training post in psychiatry in 2018 and embraced the discipline with huge enthusiam. She had a lovely bed-side manner and great listening skills. At an early stage in her career she showed a keen interest in teaching and was often to be found with medical students on the wards instilling them with her newly acquired knowledge of psychiatry. Her eagerness for learning was infectious. She had enormous potential and there is no doubt she would have contributed enormously to the field of psychiatry.

Mamoona was a calm, caring and loving person, who was intelligent, witty and full of life. Her husband, Dr Azeem Qureshi, an anaesthetist at Newham University Hospital, describes her as a remarkable woman who gave hope and joy to those around her. She was also a very good cook and a devoted mother.

Mamoona was buried in East London, her family in Pakistan being unable to attend her funeral owing to the coronavirus travel restrictions. She is survived by her husband Azeem, and their 8-year-old daughter Narmin.

